# Follicular mycosis fungoides – A report of four Indian cases

**DOI:** 10.4103/0971-5851.64257

**Published:** 2009

**Authors:** T. Rajalakshmi, Y. K. Inchara, Meryl Antony

**Affiliations:** *Departments of Pathology, St. John’s Medical College and Hospital, Bangalore - 560 034, India*; 1 *Departments of Dermatology, St. John’s Medical College and Hospital, Bangalore - 560 034, India*

**Keywords:** *Alopecia mucinosa*, *follicular mucinosis*, *follicular mycosis fungoides*

## Abstract

**Background:**

Follicular Mycosis Fungoides (FMF) is an under-recognized disease in India. Its clinical mimics include Hansen’s disease and Sarcoidosis.

**Aims:**

To describe the clinical and pathological features of FMF.

**Materials and Methods:**

All cases of FMF between January and December 2007 were retrieved. Cases of conventional epidermotropic MF with a minor follicular component were excluded. Slides were reviewed by two observers. The following criteria were assessed: degree and density of folliculotropism of lymphocytes, location of folliculotropism (infundibular / isthmic / bulbar), follicular mucin, eosinophils, granulomas, and conventional epidermotropism. Each feature was assigned a semi-quantitative grade.

**Results:**

There were four cases of FMF, with an equal gender distribution and a mean age of 17.5 years. All lesions were on the face. They presented as: hypopigmented patches (2) and erythematous plaques (2). Alopecia was seen in two cases. The clinical diagnosis was Hansen’s disease in all four, with a differential of Alopecia mucinosa / Sarcoidosis in two cases.The histological features seen were: disproportionate folliculotropism, lymphocyte tagging with haloes, follicular mucin, and nucleomegaly / convolution in all four cases, prominent eosinophils (2), epithelioid granulomas (1), eccrine infiltration (4), parakeratosis at the follicular ostia (2), and sebaceotropism (1). The infiltrate was bulbar (4) and isthmic (2). The rest of the epidermis showed no hint of conventional MF.

**Conclusion:**

The preferential features for FMF were involvement of face, dominant folliculotropism, nuclear atypia and convolution, and follicular mucin. Presence of granulomas and eosinophils necessitated exclusion of infectious causes. The absence of findings of MF in the rest of the epidermis should not deter pathologists from rendering this diagnosis.

## INTRODUCTION

Mycosis Fungoides (MF) is a cutaneous T-cell lymphoma that is thought to be uncommon in India.[1] We are cognizant of the classic histopathological features in the plaque and tumor stages of the disease. Of late, several studies have proposed effective criteria to facilitate the diagnosis of patch stage lesions, as a consequence of which early diagnosis is possible.[2,3] However, Follicular MF (FMF) continues to elude dermatologists and pathologists and it is very often missed. In this article, we present a detailed clinicopathological analysis of four cases of FMF. The clues to the diagnosis and pitfalls are discussed here.

### Aims

To describe the clinical and histopathological features of FMF.

## MATERIALS AND METHODS

All cases reported as FMF for a period of one year (January to December 2007) were retrieved. There were four such cases, which qualified by the presence of a dominant histological pattern of disproportionate folliculotropism. We excluded cases with a predominant conventional epidermotropic pattern with a focal / minor follicular infiltrate. The clinical details were obtained from the case files. Biopsy slides were reviewed by two pathologists independently. In addition to the classical findings of MF, emphasis was laid on the following histological features: degree and density of folliculotropism of lymphocytes, presence of follicular mucin (confirmed by Alcian blue - PAS stain), presence and number of eosinophils (< 5, 5–20, > 20 per section), location of folliculotropism (infundibular / isthmic / bulbar), presence of granulomas, presence of conventional epidermotropism outside the follicles. In case of granulomas, special stains for fungi and acid-fast bacilli were performed to look for an infectious agent.

## RESULTS

**Figure 1:**
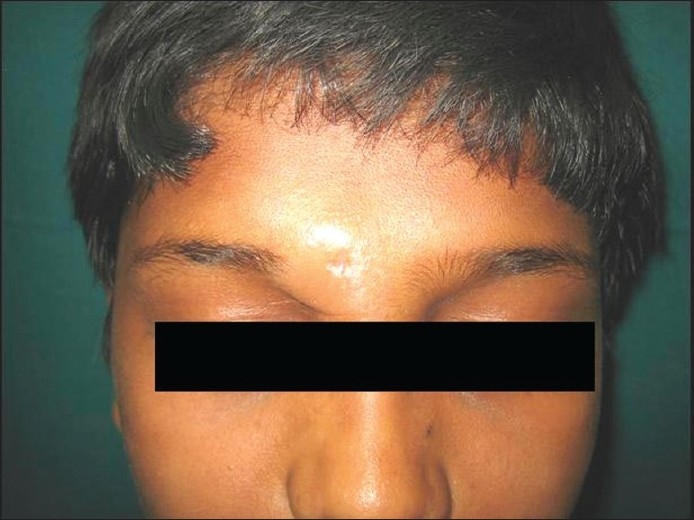
Plaque near the right eyebrow with hair loss

There were four cases of FMF over one year, with an equal gender distribution (2:2) and a mean age of 17.5 years. All the lesions were on the face. They presented as: hypopigmented patches (2) and erythematous plaques (2). Alopecia was also seen in two cases [Figure 1]. The clinical diagnosis was Hansen’s disease in all four, with a differential of Alopecia mucinosa and Sarcoidosis in two cases.

**Figure 2:**
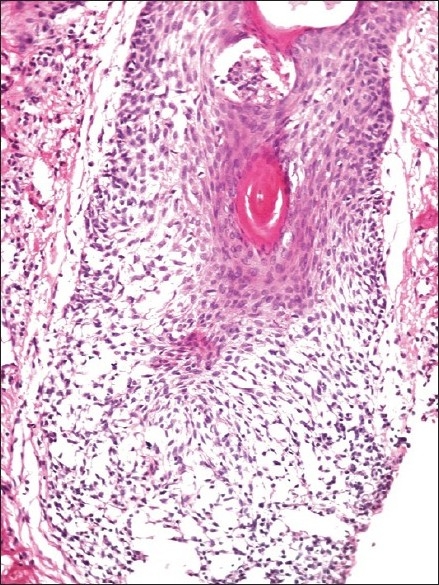
Dense folliculotropic, monomorphous lymphocytic infiltrates (H and E, × 200)

**Figure 3:**
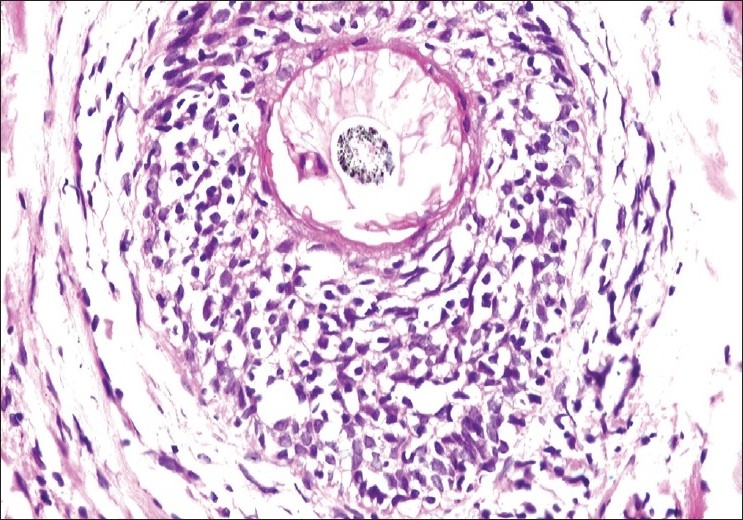
Lymphocyte tagging along the basal layer, haloes, and nuclear enlargement (H and E, × 400)

**Figure 4:**
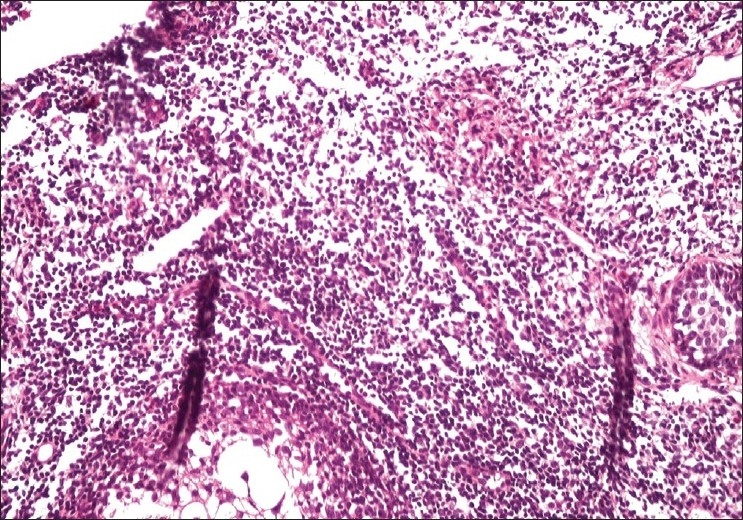
Ill-formed granuloma around the follicular units (H and E, × 200)

**Figure 5:**
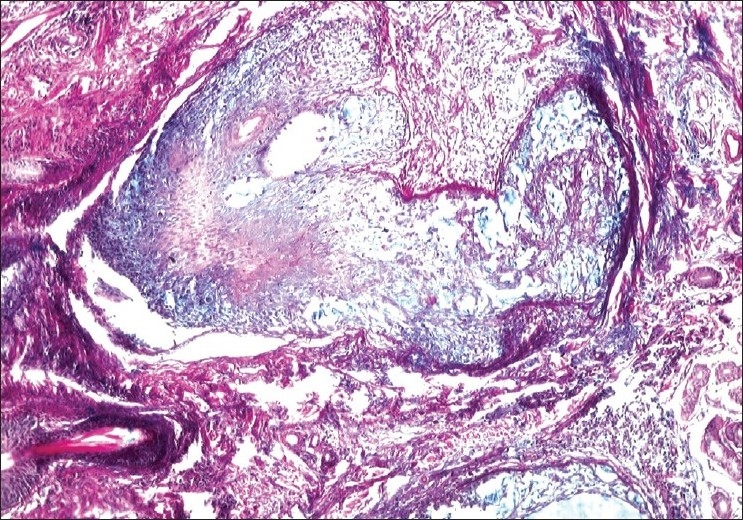
Alcian blue positive mucin within the follicles (Alcian Blue, × 200)

The histological features are depicted in Table 1. The most striking feature on biopsy, discernible even on scanning magnification, was a moderate-to-dense folliculotropic cellular infiltrate [Figure 2]. This infiltrate was populated by monomorphous lymphocytes in two cases, while the other two showed an admixture of many eosinophils (> 20 per section). There was invasion of the follicular units by these lymphocytes, with tagging along the basal layer in rows [Figure 3]. The infiltrate was seen involving the bulb of the follicle in all four cases, in addition to the isthmus in two cases. The lymphocytes showed enlargement and convoluted nuclei, surrounded by haloes. The case that showed a profusion of eosinophils also showed epithelioid granulomas centered on the involved follicles. [Figure 4] Special stains for fungi and Mycobacteria were negative. Mucin was present within the follicle in all four cases, which was highlighted by an Alcian blue stain [Figure 5].

**Table 1:** Histological features in FMF

Histological features	No. of cases (Total = 4)
Disproportionate folliculotropism	4
Location within the follicle	Bulbar (2) Bulbar + isthmic (2)
Monomorphous lymphoid infiltrate	4
Lymphocyte tagging / haloes	4
Nuclear atypia / convolution	4
Parakeratosis at follicular ostia	2
Epidermotropism	0
Pautrier’s abscesses	0
Eosinophils (> 20 per section)	2
Plasma cells	0
Epithelioid granulomas	1
Follicular mucin (Alcian blue positive)	4
Infiltration of eccrine units	4
Sebaceotropism	1
Interface dermatitis	0
Wiry collagen	0
Epidermal spongiosis (mild)	2

Infiltration of eccrine units was seen in all four cases. Marked sebaceotropism was seen in one case.

The epidermis showed focal spongiosis (two cases). It did not show features such as disproportionate epidermotropism, tagging, Pautrier’s abscesses, haloed lymphocytes, Pagetoid spread or lymphocyte atypia. Parakeratosis was noted only at the follicular ostia in two cases. There was no evidence of interface dermatitis or wiry collagen.

## DISCUSSION

Mycosis Fungoides (MF) is deemed to be rare in India.[1] A major contributing factor could be the fact that the disease is under-recognized, both by dermatologists and pathologists, especially in the early / patch stage. Thanks to the genius of Dr. Ackerman and colleagues, there are several sensitive histological criteria described that render its diagnosis possible and plausible in the patch stage itself.[2,3] While conventional ‘epidermotropic’ MF is recognized more easily, there are several clinical / histological variants that famously mimic inflammatory dermatoses.[4,5] Of these, FMF is the most significant. We did not find any reports in Indian literature, and therefore, we have attempted to profile Indian patients with FMF.

FMF commonly occurs in the head and neck region.[5] All four of our cases appeared on the face. Presentations include follicular erythematous papules, plaques, cysts, and comedone-like lesions.[6] Alopecia is common and can be seen in up to 65% of the cases.[7,8] Two cases in our series showed these features. The remaining two cases presented as hypopigmented patches, indistinguishable from Hansen’s disease clinically. Loss of sensation is very difficult to elicit from lesions on the face, and hence, one has to consider this diagnosis. The juicy, infiltrated nature of the plaques brings into play differential diagnoses such as Sarcoidosis and DLE. One striking feature in this series is the young age of our patients, that is, a mean of 17.5 years (Range: 13–27 years). The western literature reports a mean age of 55 years.[5] Lesions of MF were not seen elsewhere in the body in our series.

There seems to be divergent opinion as to the histological definition and terminology of FMF. The disease has been variously designated as Folliculotropic MF, MF with follicular mucinosis, and Alopecia Mucinosa (AM).[5,7] The last term has given rise to the maximum conjecture, with schools of belief separating the MF-associated and non-MF associated AM.[9] The same confusion also prevails over the presence of follicular mucin, with some authors claiming that mucin negates a diagnosis of MF. Flaig *et al*. have published a series of nine cases of FMF, which somewhat refines and clarifies these issues.[5] The unifying feature in all the lesions of FMF is infiltration of hair follicle epithelium by lymphocytes, causing varying degrees of damage. The lymphocytes show varying degrees of nuclear atypia. All our cases have demonstrated the above-mentioned features, with involvement of the bulbar portion of the follicle chiefly, followed by the isthmus. An important caveat here is that the biopsy should include hair follicles. If not, one may simply see a dense interstitial infiltrate and wonder about its significance. Multiple biopsies are sometimes needed for a conclusive report.

All four cases showed mild-to-moderate cytological atypia with convoluted nuclei, perinuclear haloes, and tagging along the basal layer. These were similar to the conventional MF. It is noteworthy that in the absence of atypia, FMF is difficult to establish.[5] In such an instance, the differential diagnoses to be entertained are a ‘pseudolymphomatous’ infiltrate owing to a drug or folliculitis and lymphomatoid papulosis.[10,11] The reactive germinal centers are a useful clue, as is the mixed cellular infiltrate. Clonality, although touted to be useful, does not clinch the diagnosis and might be seen in benign infiltrates.[5] A wait-and-watch policy has to be adopted in such cases.

Gerami and Guitart have described five characteristic patterns in FMF.[12] They are: basaloid folliculolymphoid hyperplasia with folliculotropism (we did not find this in our series), granulomatous dermatitis with folliculotropism, eosinophilic-follicultis-like picture with folliculotropism, dilated follicular cysts with folliculotropism, and prototypical FMF with / without follicular mucinosis. Multiple patterns can be seen in a single biopsy.

We observed a profusion of eosinophils in two cases. This mimicked eosinophilic folliculitis, which could be HIV-associated.[5] In the Indian population, one has to also keep in mind parasitic infestations, fungi, and arthropod bite reactions. Lymphocyte folliculotropism is the distinguishing factor and multiple sections needed to be examined.

We encountered folliculocentric granulomas in one case that also had many eosinophils. This represented a response to the infiltration and rupture of the follicular units, ultimately leading to alopecia. The presence of such granulomas in FMF, which clinically mimics Sarcoidosis is a major pitfall. The folliculocentricity of the granulomas and disproportionate folliculotropism serve as pointers. Ruptured follicles could also give rise to suppuration, necessitating the exclusion of an infectious etiology, which is by far the most common in our patients.[12] Special stains for microbial pathogens were negative in our case. It is important not to overcall FMF in this situation.

The most controversial criterion and terminology relate to the presence of epithelial mucin within the follicles, observed in all four cases. This is a pattern of response to follicular damage, seen in a host of conditions such as rosacea, contact dermatitis, Ofuji’s disease, Angiolymphoid hyperplasia with eosinophilia, and FMF.[5,7] These entities have been lumped under the umbrella of Alopecia mucinosa or Follicular mucinosis. It is believed that AM is an ‘abortive lymphoma’, with a propensity to progress to MF. There are studies that have attempted to differentiate between MF-associated and ‘idiopathic’ follicular mucinosis. Histology has not proved to be reliable in accomplishing this distinction.[13] Boer *et al*. have presented conclusive proof that AM is but one expression of MF and it is a lymphoma from its inception.[14] The degree and density of the infiltrate in FMF is far in excess. The same set of criteria should be used for the diagnosis of MF / FMF, regardless of mucin. The authors also recommend relinquishing the above-mentioned terms in favor of ‘MF with epithelial mucinosis’, to avoid confusion and impart specificity to the diagnosis.

Infiltration of eccrine units by lymphocytes (seen in all four cases) and sebaceotropism (one case) are seen in MF and help to exclude inflammatory mimics. They may be accompanied by acrosyringeal and ductal eccrine hyperplasia.[15] They stand proof that MF is an epitheliotropic lymphoma, rather than just an epidermotropic one.

The rest of the epidermis did not show epidermotropism / Pautrier’s abscesses. This was in concordance with the previous findings that epidermotropism was a variable feature in FMF, and was influenced by steroid use.[5] FMF could be unilesional and absence of lesions elsewhere did not preclude its diagnosis.

On immunohistochemistry, FMF comprises of CD3+, CD4+ T-lymphocytes. There is an elevated CD4:CD8 ratio within the follicular infiltrate. This may also be seen in inflammatory disorders, albeit to a lesser extent. Therefore, it is not a conclusive test and has not been performed in our cases. An increase in the CD1a+ Langerhans cell density has also been reported.[12] Demonstration of monoclonality by PCR is the only reliable ancillary test for establishing FMF, and it is not easily accessible in India. It is possible to make this diagnosis based on the predominant folliculotropic nature of lymphocytes and other accompanying histological changes in a clinically suitable lesion.

One of our cases responded to topical steroids and is currently asymptomatic, at a two-year follow-up. One patient subsequently developed patches and plaques of conventional MF over the trunk and is on PUVA therapy. The other two cases were lost to follow-up. It has been reported that FMF behaves more aggressively in comparison to conventional epidermotropic MF, and is also refractory to treatment. The Dutch Cutaneous Lymphoma group reported 36% disease progression within five years of diagnosis for FMF, in comparison to 12% for classic MF and 24% for tumor-stage MF.[16] Gerami *et al*. have demonstrated that with advanced disease (Stage IIB or more), there is no difference between the outcomes in FMF and conventional MF.[8] We need to follow-up our cases for a longer duration to comment on this aspect.

In summary, lesions of FMF preferentially involve the head and neck region, may be unilesional, and Indian patients, seem to present at a younger age. At histology, we recognize FMF by virtue of a disproportionate folliculotropic infiltrate of lymphocytes displaying varying grades of atypia, follicular mucin, and infiltration of the eccrine / sebaceous units. Eosinophils and granulomas are often seen, which mimic conditions such as pseudolymphomatous folliculits, drug reactions, and infections. These must be excluded before FMF is considered. If one is not sure initially, it is prudent to follow up these lesions and repeat biopsies. We would like to emphasize that it is not necessary for the patient to have lesions of classic MF elsewhere, or proof of epidermotropism, to establish a diagnosis of FMF. It is vital to recognize this variant as it is frequently refractory to treatment and can be undercalled as an inflammatory dermatosis.
